# Brachiocephalic Vein Reconstruction Following the Excision of a Large Suspicious Anterior Mediastinal Mass

**DOI:** 10.7759/cureus.64176

**Published:** 2024-07-09

**Authors:** Zaid Al Ghananeem, Mohamed Elsherif, Luigi Ventura, Mohammad Abuzenah, Hamza Abuzenah

**Affiliations:** 1 Vascular Surgery, Sheffield Teaching Hospitals NHS Foundation Trust, Sheffield, GBR; 2 Thoracic Surgery, Sheffield Teaching Hospitals NHS Foundation Trust, Sheffield, GBR; 3 Neurosurgery, Sheffield Teaching Hospitals NHS Foundation Trust, Sheffield, GBR; 4 Thoracic Surgery, Yarmouk University, Irbid, JOR

**Keywords:** mass, sternotomy, reconstruction, mediastinum, superior vena cava (svc) syndrome

## Abstract

Superior vena cava syndrome (SVCS) is a clinical condition characterized by signs and symptoms resulting from the blockage or narrowing of the thin-walled superior vena cava (SVC). This obstruction can lead to significant morbidity and mortality. In this case, we report a 58-year-old patient who was diagnosed with SVCS due to a massive compressing anterior mediastinal mass leading to signs and symptoms of SVCS, including shortness of breath, dizziness, palpitations, and neck swelling, which was managed surgically by excision of the mass and reconstruction of the brachiocephalic vein using a synthetic graft.

## Introduction

Superior vena cava syndrome (SVCS) occurs due to blockage or narrowing of the superior vena cava (SVC) [1-3]. The brachiocephalic vein on both sides forms from the confluence of the internal jugular and subclavian veins. It drains venous blood from the head, neck, upper limbs, and upper thorax. Major tributaries include the vertebral, internal thoracic, and inferior thyroid veins. The primary causes of SVCS are malignant obstruction (resulting from neoplastic invasion of the vein) and extrinsic compression. Extrinsic compression damages the vessel wall and alters blood flow, thereby increasing the risk of thrombosis. It is important to note that both mechanisms can coexist [1-4]. The key factor influencing the onset of SVCS is the timing at which venous restriction first occurs. Collateral circulation through the azygos vein and inferior vena cava can emerge due to the gradual progression of lesions outside the SVC causing obstruction of it. In some cases, this collateral circulation may mitigate or prevent the development of SVCS. SVCS can result from either benign or malignant extrinsic compression. Roughly 10% of SVCS cases are attributed to small-cell lung cancer, which is the most common extrinsic cause [5].

Furthermore, non-malignant factors such as aortic aneurysms contribute to extrinsic aetiologies of SVCS. Lymphoma and metastatic tumors are also implicated in SVCS [6]. Interestingly, although rare, non-Hodgkin lymphoma (NHL) can lead to SVCS, occurring in only 2-4% of NHL cases [7]. Timely and efficacious intervention targeting the malignant cause is crucial for improving prognosis. 

The level of involvement of the brachiocephalic veins determines the necessary extent of repair or reconstruction. Multiple approaches exist for reconstructing the SVC, brachiocephalic veins, or any combination of these structures. Options such as polytetrafluoroethylene (PTFE), homografts, autologous veins, and bovine or porcine pericardial tubes all serve the common goal of restoring unimpeded blood flow.

## Case presentation

**Figure 1:**
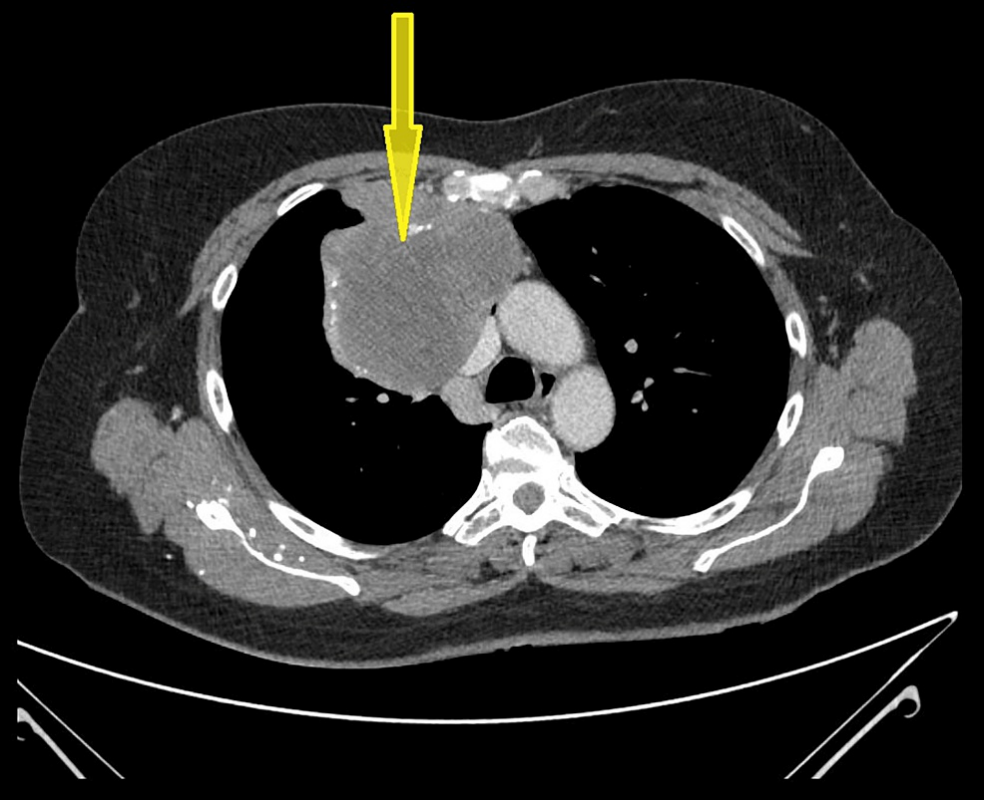
Axial-view CT scan showing the anterior mediastinal mass compressing the lower superior vena cava (SVC).

A 58-year-old fit, healthy, and non-smoker female with no remarkable past medical history presented with pain in her right shoulder. An initial X-ray revealed an incidental lesion in her right lung. Subsequently, she underwent a chest CT scan, which identified a large anterior mediastinal mass (Figure 1).

**Figure 2:**
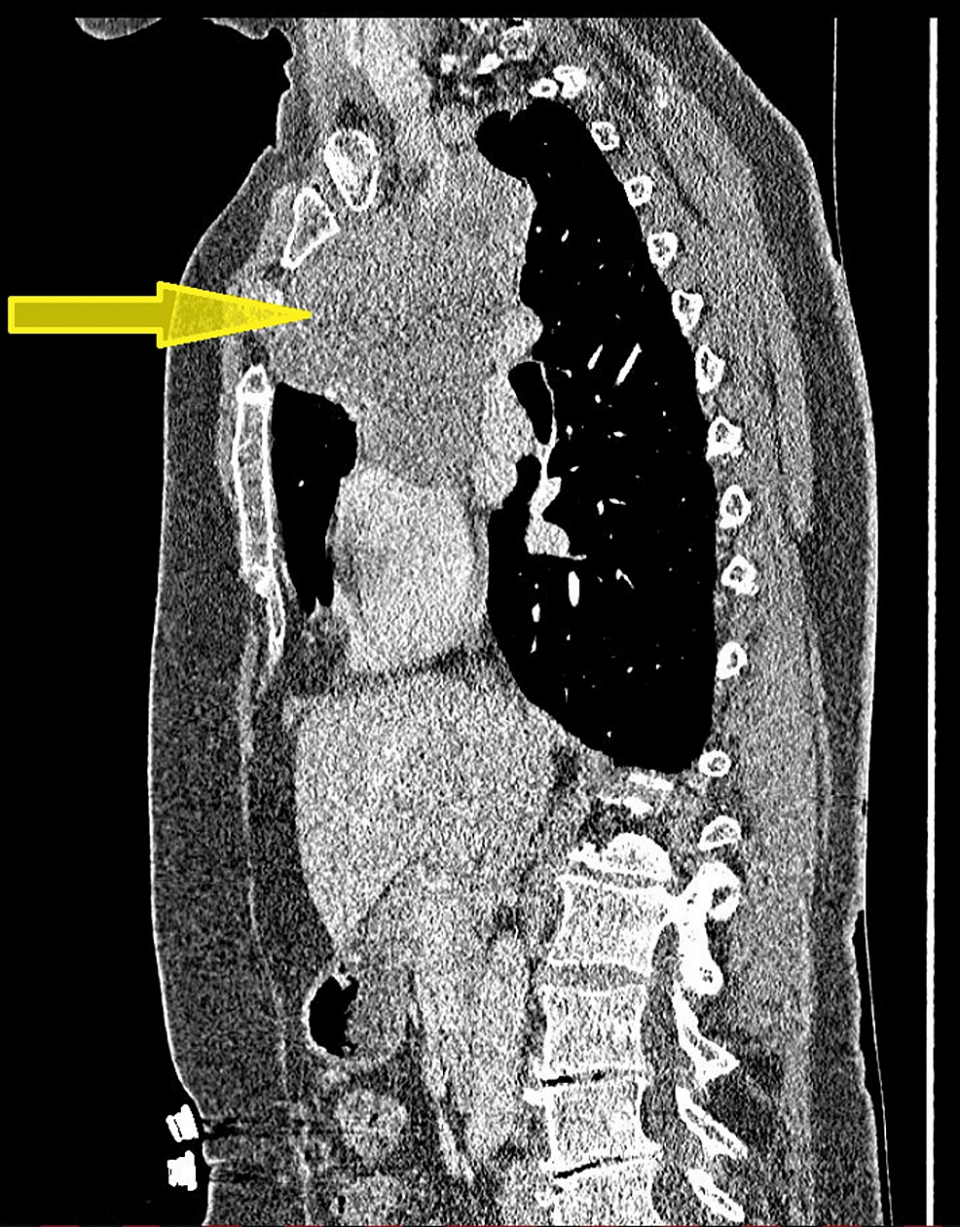
Sagittal-view CT scan showing the anterior mediastinal mass compressing the lower superior vena cava (SVC).

This mass was measuring approximately 12 cm in its largest cranio-caudal dimension (Figure 2). The mass extended retrosternally on the right side, exerting pressure and compressing the SVC. There were no other signs of vascular invasion and no associated mediastinal or supraclavicular lymphadenopathy.

A positron emission tomography (PET) scan was done, and it shows that the 125 x 88 x 72 mm right anterior mediastinal mass exhibits varying uptake, including intense avidity with a maximum standardized uptake value (SUV) of 28.2. Beyond that, there is an area of necrosis or cystic change. A CT-guided biopsy confirmed that this profile aligns with an epithelial malignancy.

The patient was consulted in the thoracic clinic. Despite being mostly asymptomatic, she experienced occasional palpitations and persistent right shoulder pain. She was advised for mass excision pending the final histopathology report and a satisfactory pre-operative assessment. Surprisingly, the histopathology report indicated that the mediastinal lesion was a breast cancer metastasis. However, due to discrepancies between the histopathology diagnosis and the clinical and radiological findings, the lesion was not classified as suggestive of metastatic breast cancer during the breast multidisciplinary team (MDT) meeting. Consequently, the thoracic surgery procedure was deemed appropriate.

In April 2024, the patient was admitted with SVCS symptoms in the form of increased pressure and swelling in the head and neck, shortness of breath (SOB), dizziness, headache, and lightheadedness. She underwent successful surgery at Sheffield Teaching Hospitals (STH), performed collaboratively by the thoracic surgery and vascular surgery teams. Preoperatively, the anesthetic inserted a left internal jugular vein (IJV) line in order to check IJV pressure to prevent any possible cerebral edema. The surgical procedure involved a median sternotomy with a right anterior cervicotomy. First, the right IJV was dissected followed by the dissection of the left brachiocephalic vein and SVC. Then, the anterior mediastinal mass was excised with en bloc resection with the vein. In addition, a thrombus was discovered in the left brachiocephalic vein, necessitating thrombectomy during the surgery. However, the left brachiocephalic vein was small, which indicates the chronicity of the compression and the clot.

**Figure 3:**
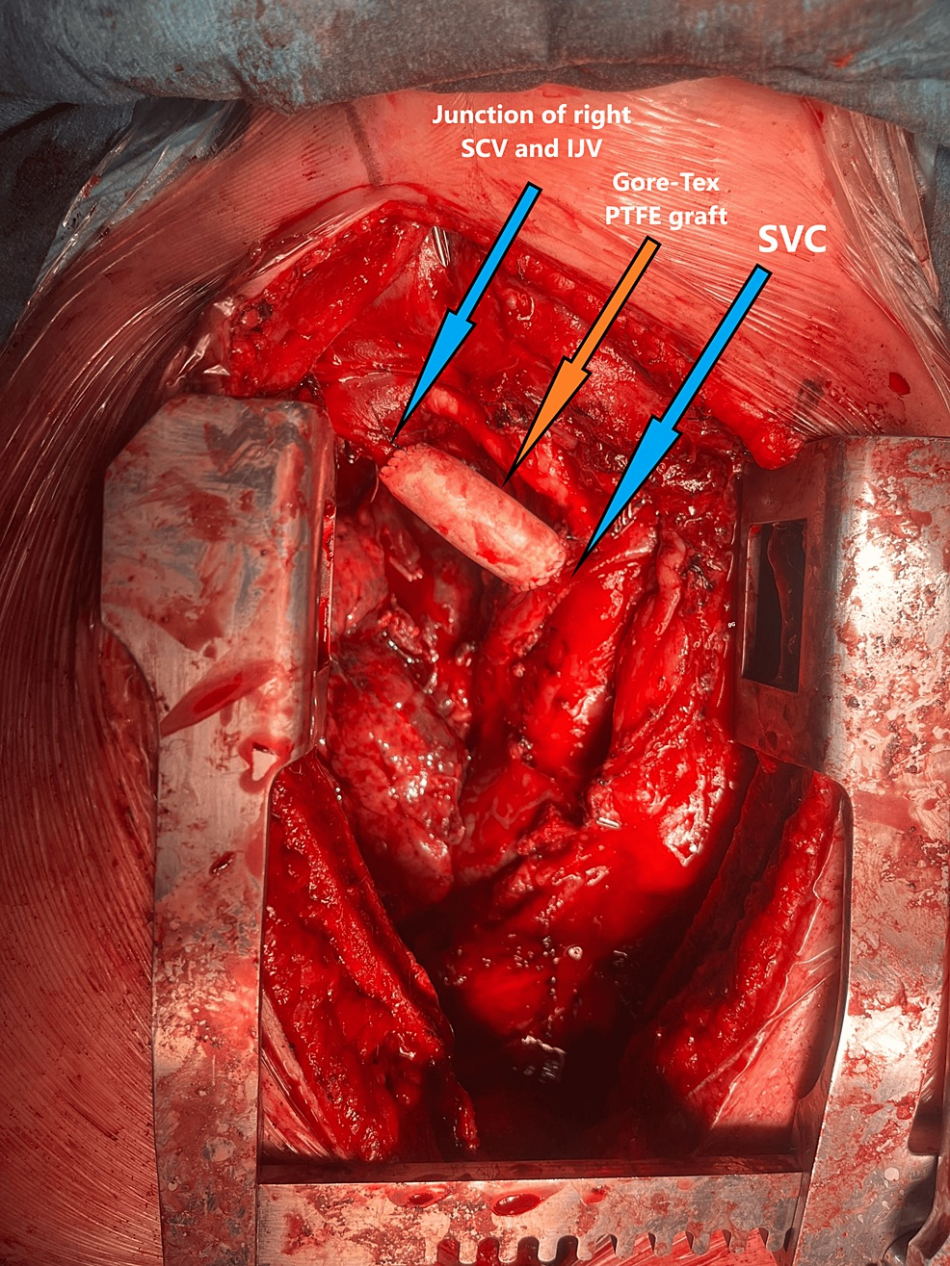
Vein bypass between the superior vena cava (SVC) and the junction of the right subclavian vein (SCV) and the internal jugular vein (IJV).

En bloc removal of the mass was made as the tumor was invading the right brachiocephalic vein with tumor emboli inside the vein and the wall was infiltrated. The reconstruction of the right brachiocephalic vein using polytetrafluoroethylene (PTFE) Gore-Tex graft 18 mm (Figure 3). Specifically, end-to-end anastomosis between the graft and the SVC above the confluence with the left brachiocephalic vein origin. Distal end-to-end anastomosis was performed between the graft and the confluence junction of the subclavian vein (SCV) and the internal jugular vein (IJV) and wedge resection of the right upper lobe in the area closest to the tumor with an Endo Gia 60 mm black tri-staple.

After the surgery, the patient began intravenous heparin, which was later switched to low-molecular-weight heparin (LMWH) during her hospitalization. Upon discharge, she continued direct oral anticoagulants (DOACs) for a duration of three months. Notably, the patient’s symptoms significantly improved following the surgery, and she is scheduled for follow-up in the clinic.

**Figure 4:**
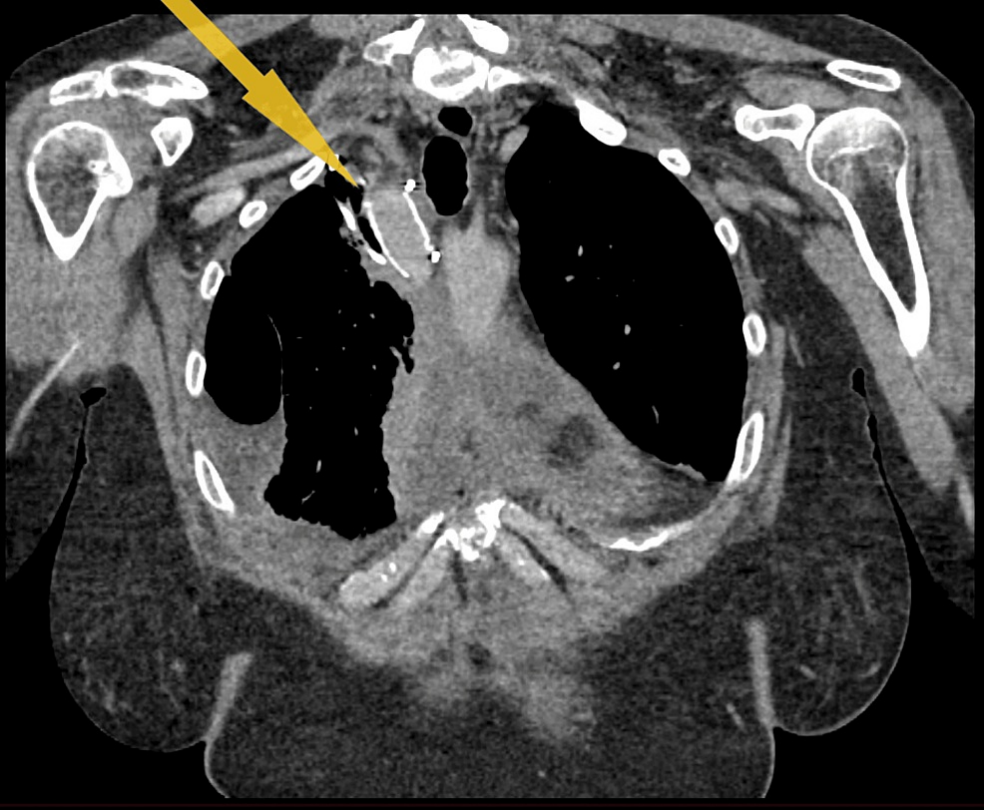
Coronal-view CT scan showing the patient's synthetic graft.

The subsequent follow-up computed tomography angiogram (CTA) scan, performed one month after the operation, revealed that the synthetic graft used for the right brachiocephalic reconstruction remains patent (Figure 4). This positive finding suggests that blood flow through the graft is unobstructed and functioning as intended.

The final histopathology report found a high-grade mediastinal non-small cell malignancy with involvement of the innominate artery and vein, with differential diagnosis including metastatic breast or urothelial carcinoma or primary thymic carcinoma.

## Discussion

This case report highlights the diagnostic complexity and underscores the significance of a comprehensive, multifaceted approach to patient management. Collaborative efforts across multiple medical teams are crucial, especially in intricate surgical procedures, to ensure favorable outcomes and maintain high standards of care.

The unique presentation of an external tumor causing SVCS in a middle-aged woman with no significant medical history emphasizes the importance of early detection and appropriate surgical intervention to prevent adverse outcomes related to SVC obstruction. This case serves as a reminder to explore all diagnostic possibilities, consider this diagnosis when typical signs emerge, and avoid delays in diagnosis and treatment. Furthermore, it underscores the pivotal role of histopathology, as biopsy results can significantly alter the management plan.

The anatomical location of the SVC and brachiocephalic veins places this venous system in a crucial region that is susceptible to tumors impacting the SVC. Mediastinal masses exhibit a diverse spectrum of lesions. The most common among these include thymoma, neurogenic tumors, and benign cysts. Together, these three categories account for 60% of patients with mediastinal masses [8]. In adults, the most prevalent mediastinal masses are primary thymic neoplasms, thyroid masses, and lymphomas [8]. The mediastinum is defined by the pleural cavities on the sides, the thoracic inlet above, and the diaphragm below. Anatomically, it is further divided into anterior, middle, and posterior compartments [9]. Anterior mediastinal tumors account for 50% of all mediastinal masses. They include conditions such as thymoma, teratoma, and lymphoma [10].

X-rays can reveal several mediastinal reflections, and their presence or distortion plays a crucial role in interpreting mediastinal abnormalities [9]. However, when evaluating a mediastinal mass, CT is the most essential diagnostic tool [11]. High-resolution multiplanar reformation images reveal the intricate anatomical connections between the tumor and neighboring structures. The excellent soft tissue contrast makes MRI an ideal modality for assessing mediastinal tumors as well [12]. Evaluating preoperative relationships with the pericardium, heart cavities, spinal cord, and vascular involvement is a common indication.

The patient’s progression from a straightforward chief complaint of right shoulder pain, which could have been attributed to common causes like muscle spasms, arthritis, or dislocation, took an unexpected turn when initial radiological scans revealed a mass that turned out to be a tumor. This case serves as an illustrative example of how systematic patient management, step by step, can lead to an accurate diagnosis and appropriate treatment.

## Conclusions

SVC reconstruction, which involves the brachiocephalic veins, presents surgical challenges due to its complexity. Factors such as patient selection, surgical strategy, suitable materials, and potential complications significantly affect prognosis. Other factors that influence the chosen approach include the surgeon’s experience, the ability to achieve proximal and distal control of the SVC or brachiocephalic veins, and the degree of vein wall involvement.
